# Integrative Analysis of Somatic Mutations in Non-coding Regions Altering RNA Secondary Structures in Cancer Genomes

**DOI:** 10.1038/s41598-019-44489-5

**Published:** 2019-06-03

**Authors:** Funan He, Ran Wei, Zhan Zhou, Leihuan Huang, Yinan Wang, Jie Tang, Yangyun Zou, Leming Shi, Xun Gu, Melissa J. Davis, Zhixi Su

**Affiliations:** 1 Ministry of Education Key Laboratory of Contemporary Anthropology, School of Life Sciences, Fudan University, Shanghai, 200433 China; 2 Institute of Drug Metabolism and Pharmaceutical Analysis and Zhejiang Provincial Key Laboratory of Anti-Cancer Drug Research, College of Pharmaceutical Sciences, Zhejiang University, Hangzhou, 310058 China; 3 Shanghai Cancer Center and Cancer Institute, Fudan University, Shanghai, 200032 China; 4 Department of Genetics, Development and Cell Biology, Iowa State University, Ames, Iowa 50011 USA; 5 Bioinformatics Division, Walter and Eliza Hall Institute of Medical Research, 1G Royal Parade, Parkville VIC, 3052 Australia; 6 Present Address: Singlera Genomics Inc, Shanghai, China

**Keywords:** Cancer genomics, Computational biology and bioinformatics

## Abstract

RNA secondary structure may influence many cellular processes, including RNA processing, stability, localization, and translation. Single-nucleotide variations (SNVs) that alter RNA secondary structure, referred to as riboSNitches, are potentially causative of human diseases, especially in untranslated regions (UTRs) and noncoding RNAs (ncRNAs). The functions of somatic mutations that act as riboSNitches in cancer development remain poorly understood. In this study, we developed a computational pipeline called SNIPER (riboSNitch-enriched or depleted elements in cancer genomes), which employs MeanDiff and EucDiff to detect riboSNitches and then identifies riboSNitch-enriched or riboSNitch-depleted non-coding elements across tumors. SNIPER is available at github: https://github.com/suzhixi/SNIPER/. We found that riboSNitches were more likely to be pathogenic. Moreover, we predicted several UTRs and lncRNAs (long non-coding RNA) that significantly enriched or depleted riboSNitches in cancer genomes, indicative of potential cancer driver or essential noncoding elements. Our study highlights the possibly neglected importance of RNA secondary structure in cancer genomes and provides a new strategy to identify new cancer-associated genes.

## Introduction

The RNA secondary structure plays a crucial role in gene regulation through affecting RNA localization, stability, splicing and translation efficiency. As most of the human genome is transcribed^1^, the structure of RNA may profoundly influence the process of post-transcriptional regulation and translation efficiency^2^. Thus, analysis of RNA secondary structure might help us to better understand its molecular and biological role in regulation.

A number of computational and experimental methods have been developed to predict RNA secondary structures or tertiary structures, which also help to identify mutations associated with RNA structures^3–7^. For example, the advent of transcriptome RNA structure probing has enabled researchers to perform transcriptome-wide characterization of RNA secondary structure^8–10^. Through the parallel analysis of RNA structure, a recent study has identified nearly 15% of all transcribed SNVs in a trio family (father, mother, and child) as riboSNitches, which altered local RNA structures. As such mutations are heritable, riboSNitches in human genome should be quite prevalent^9^. The distribution of riboSNitches varies among different elements of the transcripts. While genome-wide studies have demonstrated that riboSNitches were significantly depleted near RNA regulatory elements such as miRNA and protein binding sites^11^, recently studies have also revealed that mutations which alter local RNA secondary structures of RNA binding protein (RBP) binding sites may influence their affinity with corresponding RBP^12–16^. These findings suggest that SNVs altering RNA secondary structure may play pivotal roles in gene regulation.

RiboSNitches potentially contribute to human diseases, including cancers. It has been suggested that mutations responsible for hyperferritinemia cataract syndrome and retinoblastoma may disrupt gene expression by altering RNA secondary structure^17–19^. RiboSNitches have also been found in RNase MRP lncRNAs and these mutations may be relevant to human cartilage-hair hypoplasia^20^. Recently, researchers also found that a riboSNitch in 3′UTR of *FKBP5* could mediate susceptibility to chronic post-traumatic pain through altering the binding of miR-320a to this gene^21^. In a word, SNVs that disrupt key structural elements of a RNA can result in its dysfunction and cause human disease. As for cancer, some cancer-associated riboSNitches have been identified in non-small cell lung cancers, especially in UTRs and around miRNA binding sites^22^, and in retinoblastoma in *RB1* 5′UTR^17^.

Many previous studies have been able to discover cancer driver noncoding elements, especially in regulatory regions such as promoters and enhancers^23–27^. A recent pioneer study has predicted the functional impact of mutations based on RNA structural alterations and CADD (Combined Annotation Dependent Depletion) prediction to detect cancer-driver lncRNAs, suggesting that it might be a useful approach to detect driver noncoding elements leveraging the impact of mutations on the RNA secondary structure^24^. Compared with the secondary structure of RNA, sequence conservation is low, and may not be an effective indication of the functional importance of noncoding regions. For instance, although the sequence conservation of lncRNAs is relatively weak in primates, their secondary and tertiary structures are highly conserved^28–30^. Thus, a mutation near such structurally conserved regions is likely to disrupt biological function by altering the local structure. The identification of riboSNitch-enriched or depleted noncoding elements might facilitate the discovery of relevant genes and ncRNAs in cancer and in other diseases as well.

The role of riboSNitches in cancer genomes remains largely unexplored. Therefore, we developed the pipeline SNIPER (riboSNitch-enriched or depleted elements in cancer genomes) to predict riboSNitches and used an empirical substitution model to simulate neutral mutation processes to identify riboSNitch-enriched or depleted noncoding elements in cancer genomes. We only focused on UTRs and lncRNAs in the current study, because of the multiple indistinguishable functional effects of coding region mutations and our limited server computing power. We used this pipeline to conduct a genome-wide analysis to explore the prevalence and the possible function of noncoding riboSNitches in cancer genomes and in tumorigenesis.

## Results

### MeanDiff and EucDiff are effective approaches to detect riboSNitches

**Figure 1:**
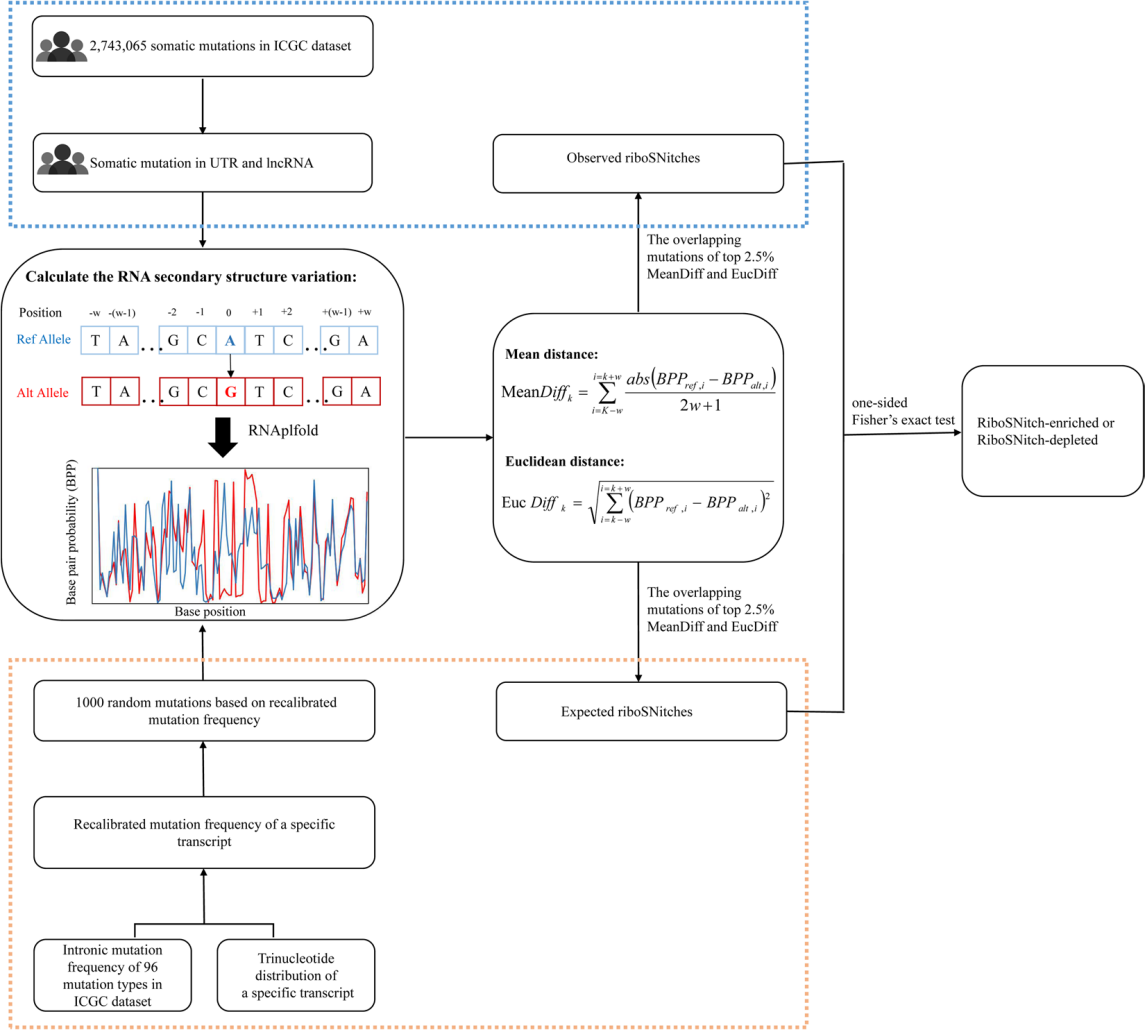
The framework of SNIPER. First, RNA secondary structure was calculated using RNAplfold for ICGC dataset and 1000 randomizations data based on intronic mutation frequency of 96 mutation types and trinucleotide distribution, separately. Then, MeanDiff and EucDiff were used to calculate the structure differences between reference and mutated sequences. Next, mutations in the top 2.5% of both MeanDiff and EucDiff were defined as riboSNitch, and in the bottom 2.5% of both MeanDiff and EucDiff were defined as non-riboSNitch. By comparing the number of observed and expected riboSNitches, riboSNitch-enriched or depleted elements can be detected.

We developed a method to detect riboSNitches. For each SNV, we replaced the corresponding reference allele with the alternative allele to generate a mutated or altered transcript (Fig. 1). Then, the RNA structure predictor were employed to reference and altered transcripts respectively, and by comparing the structural differences between the two transcripts, the impact of this SNV on RNA structure could be estimated. Rather than minimum free energy approaches, we chose the BPPM-based (Base Pairing Probability Matrix) algorithm RNAplfold to predict RNA conformation, as recommended by previous studies^31^. Here, two different methods, MeanDiff and EucDiff, were introduced to detect riboSNitches by calculating the correlation between base pair probabilites of reference and those of mutated transcripts based on RNAplfold (Fig. 1; details in the methods).

**Figure 2:**
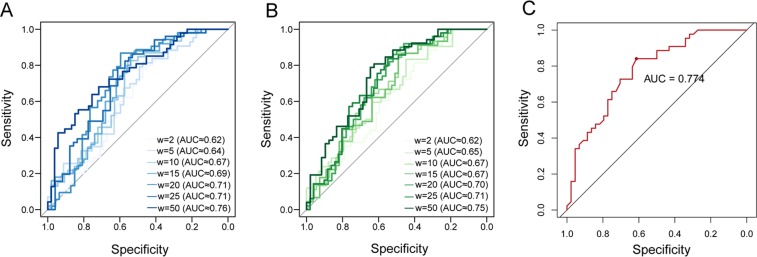
Performance of MeanDiff and EucDiff. ROC curves and AUC values were calculated for benchmark data at 5% tails of MeanDiff (**A**) and EucDiff (**B**) prediction. The color of the curves was shifted from light to dark to represent different window sizes. (**C**) The ROC curve and AUC values of the intersection of the top 2.5% MeanDiff mutations and EucDiff mutations.

To evaluate the performance of our methods, a benchmark dataset of 2,116 SNV-transcript pairs was used, including 1,058 sequences with riboSNitches and 1,058 sequences with non-riboSNitches. Each SNV and its flanking 50 bp sequence was considered as standard input for folding prediction, i.e. 101 base pairs in total^9,31^. Top and bottom 2.5% results were regarded as riboSNitches and non-riboSNitches respectively, as recommended by previous study^31^. For each method, we tested a range of window size (from 2 bp to 50 bp) when calculating BPPM value for both reference and mutated sequence. The maximum window size was set to 50 bp since the input sequences were only 101 bp. We found continuous improvements in area under the ROC curve (AUC) with increasing window size for MeanDiff and EucDiff, and the two methods showed comparable performance (Fig. 2A,B).When the window size was set to 50 bp, the ROC curves illustrated a slightly better performance of MeanDiff (AUC = 0.76) than EucDiff (AUC = 0.75). Comparing with a previous study using SNPfold (AUC = 0.736 at 5% tails)^31^, our methods with a window size of 50 bp showed a little improvement (Table 1).

**Table 1:** The AUC values in different window size using MeanDiff, EucDiff and SNPfold.

	w = 2	w = 5	w = 10	w = 15	w = 20	w = 25	w = 50
MeanDiff	0.62	0.64	0.67	0.69	0.71	0.71	0.76
EucDiff	0.62	0.65	0.67	0.67	0.70	0.71	0.75
SNPfold	NA	NA	NA	NA	NA	NA	0.736

^*^NA represents the results not provided by Corley *et al*^31^.

Considering DNA transcription is accompanied by RNA folding, we enlarged the window size to 200 bp to allow for better measurement of possible influence of RNA structure on transcription^32^. In the following analysis, to improve specificity, we determined mutations in both top 2.5% results of MeanDiff and EucDiff as riboSNitches and similarly in both bottom 2.5% results of MeanDiff and EucDiff as non-riboSNitches. Using this criterion, the AUC value increased to 0.774 (Fig. 2C), and it was used in the following analysis.

### RiboSNitches are more likely to be pathogenic variants in cancer genomes

**Figure 3:**
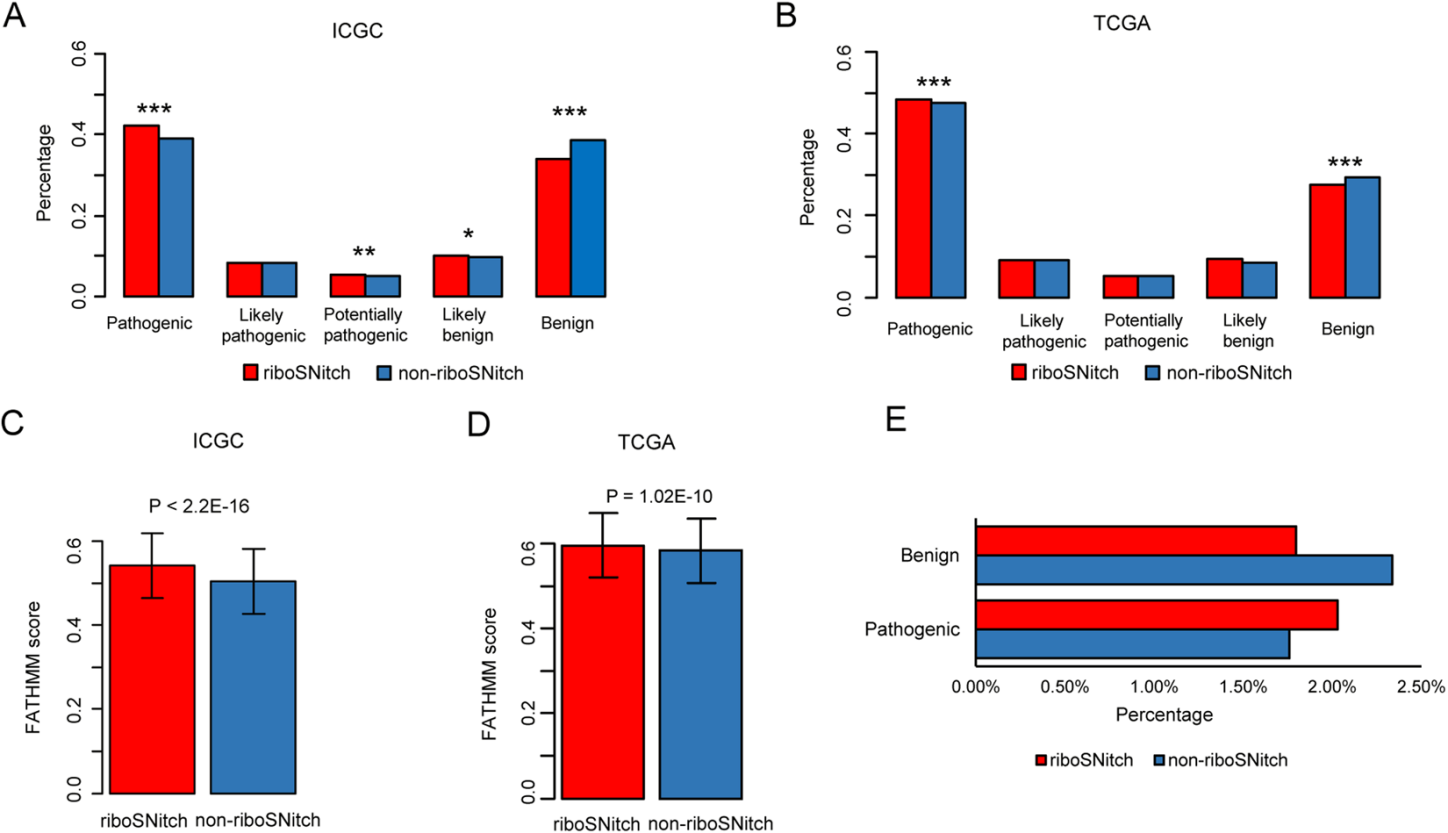
The different functional effects of riboSNitches and non-riboSNitches. (**A**) Functional consequences of riboSNitch (red) and non-riboSNitch (blue) in the ICGC dataset. (**B**) Functional consequences of riboSNitch (red) and non-riboSNitch (blue) in the TCGA dataset. We divided all the mutations into 5 categories based on FATHMM scores. The *P* value was calculated by the Chi-square test. (**C,D**) FATHMM score distribution of riboSNitches and non-riboSNitches in the ICGC and TCGA dataset. The *P* value was calculated by the Mann-Whitney test. (**E**) The different functional effects of riboSNitch and non-riboSNitch in benign and pathogenic variants. The *P* value was calculated by the Chi-square’s test.

To determine whether riboSNitches and non-riboSNitches have different functional impacts, we firstly detected riboSNitches in somatic mutations that we collected from TCGA (The Cancer Genome Atlas), ICGC (International Cancer Genome Consortium) and other previous publications^33,34^ (the results are summarized in Supplementary Table S1). Then, we predicted the functional impact scores of somatic mutations using FATHMM-MKL^35^. Given that predicted scores are continuous, we divided them into 5 bins, which were benign, likely benign, potentially pathogenic, likely pathogenic and pathogenic (higher score indicates more pathogenic). We found that the predicted scores of riboSNitches and non-riboSNitches were distributed in all 5 bins with the most significant distribution differences in benign and pathogenic bins (Fig. 3A,B). Overall, the FATHMM scores of riboSNitches are higher than those of non-riboSNitches (Fig. 3C,D). These results imply that somatic mutations that alter RNA secondary structure are more likely to be pathogenic.

To validate this conclusion, we collected a total of 91,183 pathogenic variants and 79,090 benign variants from the ClinVar^36^, UniProt^37^ and Human Gene Mutation Database (HGMD)^38^, to determine whether the riboSNitches constitute a larger proportion of pathogenic variants than non-riboSNitches. As most of the mutations were from normal human samples, we used the MeanDiff and EucDiff cutoff of the 1000 Genome Project to identify riboSNitches and non-riboSNitches in this dataset. As shown in Fig. 3E, pathogenic variants tended to be riboSNitches, and benign variants tended to be non-riboSNitches (*P* = 2.87E-05, Chi-squared test). Thus, we used these variations which were already known as benign or pathogenic to reconfirm the conclusion that riboSNitches were more likely to be pathogenic.

### Features of riboSNitches in the cancer genomes

After dividing somatic mutations into the six substitution subtypes (C > A, C > G, C > T, T > A, T > C, T > G), we found that the value of MeanDiff or EucDiff varied between them, suggesting that different substitution subtypes might have different impacts on the RNA secondary structure (Supplementary Figs S1–S4). Interestingly, C > G mutations were inclined to have a greater impact on the RNA secondary structure (according to the results of MeanDiff and EucDiff) compared with the other mutation types in both the ICGC and TCGA datasets. As for the location, mutations in 5′UTRs had overall higher scores than mutations in 3′UTRs, lncRNAs and protein-coding regions (*P* < 2.2e-16 for all comparisons, Mann-Whitney test). Intriguingly, more than 80% of the somatic mutations in 5′UTRs were found to be substitutions of GC pairs. One possible explanation is that there are more conserved RNA structures in 5′UTRs, which may imply more functional elements. Because the structure of GC pairs are more stable than AT pairs, this result also suggested that a variety of 5′UTR mutations of a GC pair in the cancer genomes may disrupt the stability of the local RNA structure.

To determine whether riboSNitches are depleted in functional regions in cancer genome, we collected RBP binding data from CLIPdb^39^ and miRNA binding targets with high confidence from the TargetScan and miRanda dataset^40,41^. In comparison to non-riboSNitches, riboSNitches were enriched around miRNA binding sites (*P* = 5E-21, one-sided Fisher′s exact test), suggesting that miRNA binding targets might be under positive selection in cancer through altering the RNA secondary structure. In contrast, riboSNitches were significantly depleted around RBP binding sites (*P* = 1.79E-07, one-sided Fisher’s exact test) in the cancer genome, consistent with a previous study of a trio family^9^, suggesting that RBP binding targets were under purifying selection in cancer genomes to maintain a constrained structure. Those results are consistent with the knowledge that RNA secondary structure plays an essential role in RNA regulation, especially in the interactions with miRNAs and RNA-binding proteins. Therefore, we suggest that further research should be conducted to discover whether the expression of these riboSNitch-enriched or depleted genes is regulated by altered RNA secondary structure in cancer.

### A computational framework for predicting riboSNitch-enriched or riboSNitch-depleted genes

To explore the potential biological function of riboSNitches in cancer development, we developed a computational pipeline called SNIPER to identify riboSNitch-enriched or depleted genes based on the dataset of cancer somatic mutations in noncoding region (Fig. 1). We hypothesized that riboSNitch-enriched genes would display an excess of riboSNitches compared with expectation, demonstrating that these genes have undergone positive selection for RNA shape during cancer progression, which could be regarded as an evolutionary process. To find those riboSNitch-enriched genes, we used MeanDiff and EucDiff to tally the number of riboSNitches in ICGC and previous studies at first. Then, we constructed a neutral mutation model based on the cancer intronic mutation profile and the tri-nucleotide context of each transcript to calculate the expected number of riboSNitches as described in Methods. Finally, through the comparison of observed riboSNitch ratio with the simulated result, we can identify riboSNitch-enriched or depleted genes (Fig. 1). Unlike previous methods, we used intronic instead of exonic mutation rate to simulate expected number of riboSNitches, and thus riboSNitch-enriched genes detected by SNIPER could be regarded as positively selected genes in cancer. As shown above, riboSNitches were more likely to be pathogenic, so we conjectured that these riboSNitch-enriched genes might play a nonnegligible role in cancer progression. On the other hand, riboSNitch-depleted genes could be structurally conserved genes in cancer, namely essential genes and could play a fundamental role in cancer process. Therefore, our approach may help us to identify potentially cancer-associated genes involved in tumorigenesis and development.

### Identification of riboSNitch-enriched elements to find cancer-related genes

To detect cancer-related candidate genes, our method was applied to the ICGC dataset in UTRs to comprehensively discover riboSNitch-enriched or depleted elements of protein-coding genes. The alteration of structure of UTRs might impact gene expression through altering miRNA or RBP binding, and thus contributes to oncogenesis. In our analysis, both MeanDiff and EucDiff were used to measure the impact of mutations on RNA secondary structure based on the results computed by RNAplfold, and the parameter was set as recommended^32^. RiboSNitches were identified as the intersection of the top 2.5% mutations of MeanDiff and EucDiff. Finally, statistical significance was calculated with Fisher’s exact test and corrected using the Benjamini and Hochberg method^42^. For protein-coding genes, we only detected cancer-related functional candidate UTRs because the RNA structure of the 3′UTRs and 5′UTRs are crucial for gene regulation and translational stability. Coding region mutations were excluded for their complex effects, and the effect of altering the RNA secondary structure might be masked by other influences.

**Figure 4:**
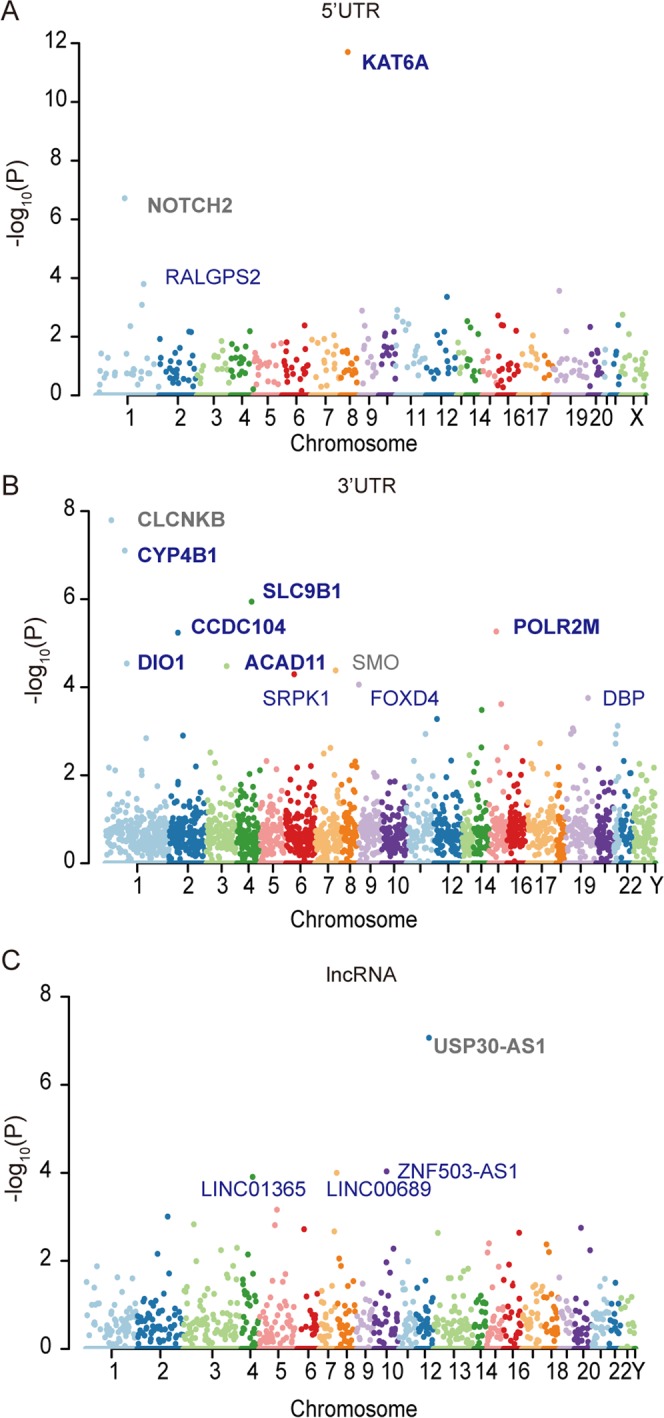
RiboSNitch-enriched elements in the cancer genome. A Manhattan plot representing RiboSNitch-enriched 5′UTRs (**A**), 3′UTRs (**B**), and lncRNAs (**C**) with the most significant *P* values. All the identified elements with an FDR < 0.2 are listed in the plot. Genes in bold represent an FDR < 0.05. Genes in blue indicate that this gene was identified as a cancer-specific enriched element.

Firstly, we applied our method to 5′UTRs to discover putative cancer driver elements. Here, for the genes with riboSNitch-enriched 5′UTRs, we found 224-fold enrichment of Cancer Gene Census (CGC) genes over random expectation (*P* < 2.2e-16, Chi-squared test). In this case, the 5′UTRs of *KAT6A* and *NOTCH2* were identified at a q-value cutoff of 0.05, and both genes were discovered in the CGC, and are regarded as known cancer genes (Fig. 4A). *NOTCH2* is regarded as an oncogene, and plays an essential role in cancer signaling pathways^43^. *KAT6A* is a lysine acetyltransferase gene that has been shown to be involved in cell growth of luminal breast cancer in a previous study^44^. In addition, *RALGPS2* was identified as a putative driver when reducing the cutoff of q-value to 0.2, and *RALGPS2* is involved in cell survival and associated with the cell cycle in lung cancer cells^45^. We found that *NOTCH2*, *KAT6A* and *RALGPS2* are all potential cancer driver genes, demonstrating that this method can help us to find RNA structure-related cancer driver elements.

Next, we also employed our approach to identify riboSNitch-enriched elements in 3′UTRs. As in the case of 5′UTRs, 7 genes with riboSNitch-enriched 3′UTRs were identified using SNIPER, including *CLCNKB*, *CYP4B1*, *SLC9B1*, *CCDC104*, *POLR2M*, *ACAD11* and *DIO1*, at the q-value cutoff of 0.05 (Fig. 4B). *CYP4B1* is a cytochrome enzyme, which has been found to be highly expressed in bladder tumor patients^46^. *SLC9B1* is a Na+/H+ transporter, which contributes to the maintenance of cellular homeostasis^47^. *POLR2M*, the RNA polymerase II subunit M, plays a crucial role in gene transcription, and known as a candidate driver gene involved in the progression of prostate cancer^48^. *ACAD11* is a gene in the acyl-dehydrogenase family, which is involved in cell survival and plays a key role in the pro-survival function of *TP53*^49^. The *DIO1* gene encodes a type I iodothyronine deiodinase, an important regulator of cell proliferation, differentiation and metabolism^50^. Additionally, the 3′UTRs of *SMO*, *SRPK1*, *FOXD4* and *DBP* were also identified as riboSNitch-enriched elements when reducing the cutoff of q-value to 0.2. Of these, *SRPK1* has been shown to have a tumor-suppressive effect and is a candidate driver gene^51,52^.

To determine whether the elements identified above are cancer-specific riboSNitch-enriched elements, we compared the observed elements in the cancer genomes to those in the germline dataset. We found that two riboSNitch-enriched 5′UTRs were cancer-specific, which were *KAT6A* and *RALGPS2*. All the 3′UTRs identified above were found to be cancer-specific riboSNitch-enriched elements, except for *CLCNKB* and *SMO*. These results suggest that cancer-specific riboSNitch-enriched elements might have the potential to be cancer-related functional elements or putative driver elements.

Last, for lncRNAs, at a q-value cutoff of 0.05, only *USP30*-*AS1* showed a significant enrichment for riboSNitches using our method, but it is not a cancer-specific lncRNA. When relaxing the q-value cutoff to 0.1, *LINC01365*, *ZNF503*-*AS1* and *LINC00689* were identified as riboSNitch-enriched, among which *ZNF503*-*AS1* and *LINC00689* were cancer-specific (Fig. 4C). As for *ZNF503*-*AS1*, it is intriguing that it can promote the proliferation and migration of pigment epithelium by regulating *ZNF503*, and expression of *ZNF503*-*AS1* has been shown to be a prognostic indicator for lung squamous cell carcinoma^53,54^. From FuncPred, a website for the prediction of lncRNA function^55^, we found that three of the lncRNAs (*USP30*-*AS1*, *ZNF503*-*AS1* and *LINC00689*) predicted above were related to the cancer process with FDR < 0.05^55^.

Additionally, considering that less than 2% of somatic mutations were identified as riboSNitches under the criterion of the intersection of the top 2.5% mutations of MeanDiff and EucDiff, we relaxed the cutoff of MeanDiff and EucDiff to 5%, 10% and 20%. All the riboSNitch-enriched elements identified by our approach at different cutoffs were listed in Supplementary Table S2.

### Identification of riboSNitch-depleted elements to find structural conserved elements in cancer

**Figure 5:**
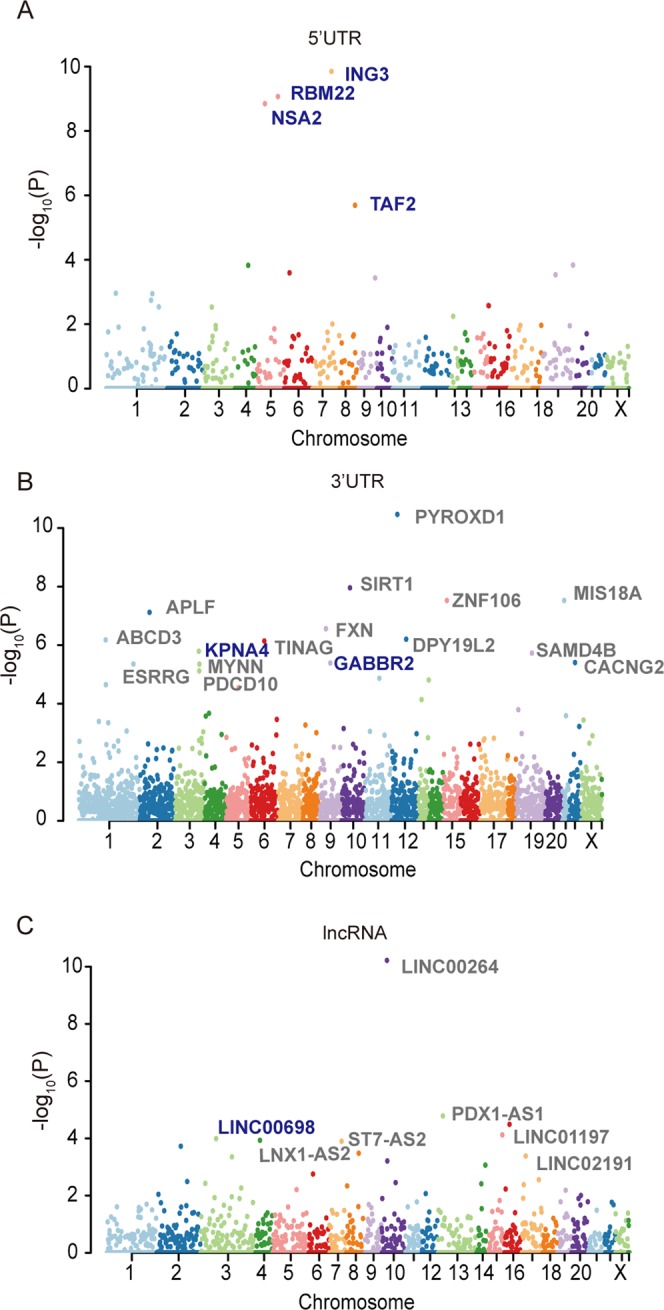
RiboSNitch-depleted elements in the cancer genome. A Manhattan plot representing RiboSNitch-depleted 5′UTRs (**A**), 3′UTRs (**B**), and lncRNAs (**C**) with the most significant *P* values. All the identified elements with an FDR < 0.05 are listed in the plot. Genes in blue indicate that this gene was identified as a cancer-specific depleted element.

Our approach can also be used to predict riboSNitch-depleted elements in cancer genomes. These elements might be essential for cancer due to their constrained RNA secondary structure. We identified riboSNitch-depleted elements, including 5′UTRs, 3′UTRs and lncRNAs (Supplementary Table S3). Four riboSNitch-depleted 5′UTRs (*ING3*, *RBM22*, *NSA2* and *TAF2*) were detected at the q-value cutoff of 0.05, and all of these elements were cancer-specific (Fig. 5A). We also discovered 22 riboSNitch-depleted 3′UTRs, but only two of them (*KPNA4* and *GABBR2*) were identified as cancer-specific (Fig. 5B). Among the six cancer-specific riboSNitch-depleted elements, five (*ING3*, *RBM22*, *NSA2*, *TAF2* and *KPNA4*) were found to be conditionally essential in cancer cell lines in the OGEE v2 database^56^. Additionally, seven riboSNitch-depleted lncRNAs were identified at the q-value cutoff of 0.05, but only *LINC00698* was cancer-specific (Fig. 5C). Our results imply that cancer-specific riboSNitch-depleted regions might be potentially cancer essential elements.

## Discussion

Our study provides a new strategy to investigate cancer somatic mutations in noncoding region from the perspective of RNA shape, and it suggests the importance of RNA secondary structure in cancer possibly by regulating the expression of genes. To our knowledge, this is the first comprehensive study to analyze riboSNitches in somatic mutations across cancer genomes based on the two main international cancer genomics repositories. In our analyses across mutations in cancer genomes, different mutation subtypes exhibited different impacts on RNA structure, and the riboSNitches were enriched around binding targets of miRNAs and depleted around those of RBPs. Those results suggest that some somatic mutations have the potential to profoundly influence the RNA secondary structures, and may further regulate gene expression^11,13,21^.

A myriad of experimental technologies has been developed to probe and analyze RNA structure, yet accurately characterizing RNA structure is still extremely difficult for the high dynamics of RNA *in vivo* and *in vitro*. Moreover, diverse computational approaches have been designed to predict the impact of a single mutation by integrating empirical data. It should be noted that predicting riboSNitches basing on experimental data was more accurate than computational methods^57^. However, for lack of experimental data of the cancer genome, it is acceptable to use computational methods to predict riboSNitches among cancer somatic mutations. In SNIPER pipeline, we detected riboSNitches using RNAplfold and a combination of MeanDiff and EucDiff, and found that the performance of our *in silico* method was better than SNPfold, which has been proven as one of the best methods listed in a previous study^31^. Though it is viable to use a computational approach to determine riboSNitches in the cancer genome, further experiments are needed to explore the functional relevance of variant-induced RNA structural alterations and their biological mechanisms responsible for disease phenotype.

In the present study, we found that riboSNitches were more likely to be pathogenic and may increase the risk of disease. Based on this, we proposed a new pipeline SNIPER to detect riboSNitches and to identify riboSNitch-enriched or depleted noncoding elements in cancer genome. First, we constructed an expected riboSNitch distribution under a neutral mutation model based on the cancer intronic mutation profile and the tri-nucleotide context of each transcript. Then, by comparing the observed riboSNitch ratio with expected, riboSNitch-enriched or riboSNitch-depleted elements could be predicted. Given the use of intronic mutation rate as a neutral background model in our method, we could detect elements under positive selection in cohorts of tumors. As shown in our results, riboSNitch-enriched UTRs could be potential cancer drivers, and riboSNitch-depleted UTRs were prone to be essential genes in cancer, especially cancer-specific ones. In addition, we identified several cancer-related lncRNAs, and the actual function of these lncRNAs in cancer progression needs to be further investigated. Above all, our method allowed us to find riboSNitch-enriched and riboSNitch-depleted noncoding elements in the cancer genome and could help us to identify more cancer driver and essential genes.

Previous methods that have been designed to identify cancer driving elements in noncoding regions tended to discover positive selection signals by comparing mutation rates between target sequences and corresponding flanking regions in noncoding sequences, especially for regulatory elements^24^. In our study, we developed a new method to identify positive selection signals by detecting the relative impact of somatic mutations located in noncoding regions on the RNA secondary structure. Although most of the human genome can be transcribed, we only focused on changes of RNA secondary structure of UTRs and lncRNAs, resulting in only a small number of mutations. Despite this limitation, SNIPER was able to identify regions significantly enrich or deplete riboSNitches in cancer genomes, and we effectively identified several candidate driver and essential elements.

Next-generation sequencing technologies have enabled genome-wide analysis of variations in human genomes, greatly enhancing our understanding of RNA structure-related variations. With the amount of cancer genome sequencing data accumulates, we can further analyze riboSNitch-enriched or depleted elements for each cancer type. In the meanwhile, although many studies have been conducted to predict potentially functional lncRNAs^55,58,59^, the molecular functions of these lncRNAs remain to be explored.

Given the complexity of coding region mutations and the limited computing power of our servers, our analysis mainly focused on the noncoding regions, namely UTRs and lncRNAs. Providing an initial analysis of riboSNitches in the cancer genome, we found that they are more likely to be pathogenic variants. Additionally, we also pave the way to explore potentially functional noncoding regions in the cancer genome from the perspective of RNA secondary structure. Although we have highlighted the potential effect of riboSNitches in the cancer cohort, it remains a challenge to validate whether such mutations are involved in tumorigenesis or play a role in post-transcriptional regulation and gene translation in cancer.

## Materials and Methods

### Datasets

The majority of cancer somatic mutation data used in this study were obtained from the ICGC and TCGA data portal. We also retrieved somatic mutations of 25 whole-genome sequenced melanomas^33^ and 100 whole-genome sequenced gastric cancers from previous studies^34^. The germline mutation dataset was retrieved from the 1000 Genome Project phase 3 data^60^.

First, we excluded all small insertions and deletions, and only single-nucleotide variations were retained for further analysis. Then, all the coordinates of mutations based on hg38 were lifted to hg19 using the UCSC liftOver toolkit^61^. To remove SNVs with low confidence, both cancer somatic mutations and germline mutations from the 1000 Genome Project were filtered against the hg19 signal artifact blacklist; the merged blacklist was downloaded from the Broad Institute (https://personal.broadinstitute.org/anshul/projects/encode/rawdata/blacklists). This list is a comprehensive collection of signal artifact blacklist regions in hg19, which included regions with abnormal mapping, repeat elements, and an enrichment of ultra-high frequency artifacts.

We used FATHMM-MKL^35^ to predict the potential effects of riboSNtiches and non-riboSNitches. According to the pathogenic score, we divided all the variants into 5 categories: benign (score ∈ [0, 0.2], likely benign (score ∈ (0.2, 0.4]), potentially pathogenic (score ∈ (0.4, 0.6]), likely pathogenic (score ∈ (0.6, 0.8]) and pathogenic (score ∈ (0.8, 1]).

The miRNA-target interactions were obtained from the TargetScan (release 7.1) and miRanda-mirSVR (August 2010 release)^40,41^. Only binding sites with high confidence were selected in our analysis. For TargetScan, conserved targets or targets of conserved miRNA families with PCT ≥ 90 were used. For miRanda, the conserved miRNAs with a high mirSVR score (cutoff was set to 1) were used. In addition, we collected all the CLIP-seq data for the HeLa cell line from CLIPdb^39^, which included the interaction regions with different RBPs detected by PiRaNhA^62^.

The known cancer genes were retrieved from CGC of COSMIC (Catalogue of Somatic Mutations in Cancer) tier 2^63^, and the cancer-related lncRNAs were downloaded from the Lnc2Cancer database^64^.

### Gene annotation

The coordinates of all transcripts were obtained from the ENCODE website (https://www.encodeproject.org/); specifically, GENCODE^65^ release 19 annotation in GTF format was downloaded at https://www.gencodegenes.org. From the GTF file, genes were assigned to protein coding genes (PCGs), pseudogenes, lncRNAs and other small noncoding genes by the “gene_type” catalogue. For more accurate annotation, we obtained the human protein-coding gene list and long noncoding RNA list from HGNC^66^. Finally, a total of 19,035 PCGs and 3,435 lncRNAs were included in our analysis.

It is noteworthy that one gene may generate multiple transcripts by alternative splicing with different RNA secondary structures. To reduce such uncertainty, one principal transcript of each gene was selected for RNA structure prediction. To obtain the principal transcript, we carried out the following steps. (1) The multi-transcript protein-coding genes were first ranked by annotating the principal splice isoform (APPRIS) level, which provides a reliable classification scheme for the transcript isoforms of the alternatively spliced genes in the human genome^67^. The APPRIS level ranges from 1 to 5, with 1 being the most reliable. (2) If transcripts of a gene were ranked equivalently, then the transcript with the CCDS ID was regarded as the more reliable one^68^. (3) In addition, the annotation level of transcripts was ranked from 1 to 3, with 1 being the most stable. (4) If the principal transcript could not be selected from all the above-described methods, the longest was regarded as the principal transcript. Thus, the principal transcripts were generated in the following priority order: APPRIS > CCDS > transcript level > transcript length.

After selecting the principal transcript of each gene, the mutations that passed the filtering were annotated. Additionally, we only considered the RNA secondary structure of mature transcript in the present study. Finally, we found 3,332,314 unique cancer somatic mutations from TCGA, ICGC and previous studies^33,34^ and 1,917,818 germline mutations from the 1000 Genome dataset.

### RNA secondary structure prediction and riboSNitch detection

RNAplfold (http://www.tbi.univie.ac.at/RNA/) can generate an average base pair probability for each site for a given sequence. With the output base pairing probability matrices, it is straightforward to detect the difference in RNA structure between the wild-type and mutant. Therefore, we used RNAplfold, which is a locally stable secondary structure prediction toolkit of the ViennaRNA package^69^, to calculate local RNA secondary structures.

RiboSNitches are defined as SNVs that have a great impact on the local RNA secondary structure^9^. To identify the impact of a given somatic mutation, we can calculate the RNA structure alteration between the tumor sequence and paired normal sequence. For lack of raw sequencing data and control the effects of other confounding factors such as genetic variations in each sample, we assumed that the normal sequences are identical to the reference genome and the tumor sequences are the same except for the mutation. Reference sequences of each transcript were extracted by BEDTools getfasta using the GENCODE v19 annotation^70^. The corresponding tumor sequences were obtained by replacing the reference base with the mutated one. Then, RNAplfold was applied to both normal and cancer sequences to predict the base pair probabilities at each site. In our study, we only predicted the RNA secondary structure of mature transcripts, and intron sequences were excluded.

After calculating the RNA secondary structure variation, we computed the differences in base pair probabilities between reference and mutated sequences using MeanDiff and EucDiff (Fig. 1). Of note, the structural alterations were not restricted to a single base, and thus we calculated the alteration of base pair probability in a *w* bp window size around the mutation site. The equations for the MeanDiff and EucDiff are as follows:

1 $$MeanDif{f}_{k}=\sum _{i=k-w}^{i=k+w}\frac{abs({{\rm{BPP}}}_{ref,i}-{{\rm{BPP}}}_{alt,i})}{2{\rm{w}}+1}$$

2 $$EucDif{f}_{k}=\sqrt{\sum _{i=k-w}^{i=k+w}{({{\rm{BPP}}}_{{\rm{ref}},i}-{{\rm{BPP}}}_{{\rm{alt}},i})}^{2}}$$

where *k* is the position of the mutation in the transcript, *w* is the window size, and BPP_ref, i_ and BPP_alt, i_ represent the *i* th base pair probability of the reference and mutated sequence, which were computed using RNAplfold. According to previous study^32^, we set the parameter of window size as 200 bp to predict the probabilities of local RNA secondary structure. In order to improve the accuracy of prediction, we defined riboSNitch as the SNVs belonging to the intersection of top 2.5% MeanDiff and EucDiff, and non-riboSNitch as it belonging to both bottom 2.5%.

We further mapped all the riboSNitches and non-riboSNitches to the binding sites of miRNA and RBPs. In our analysis, mutations located in or around binding sites (±~20 bp) were determined to have the potential to influence the binding of miRNA and RBP.

### Evaluation of the performance of MeanDiff and EucDiff using the benchmark dataset

To evaluate our methods and determine a proper way to compute RNA structure alterations due to somatic mutations, we used a benchmark dataset of identified riboSNitch and non-riboSNitch sequences, which were previously detected by parallel analysis of RNA structure method. The dataset, including 1058 riboSNitch and 1058 non-riboSNitch with sequences of 101 bp, was retrieved from a previous study^31^. RNAplfold was used to calculate the RNA structures for all sequences, and the differences in base pair probabilities of mutations were calculated using MeanDiff and EucDiff with different window size of 2 bp, 5 bp, 10 bp, 15 bp, 20 bp, 25 bp and 50 bp (the maximum window size allowed for the given sequences). As recommended, the tail 5% of MeanDiff or EucDiff were regarded as riboSNitches and non-riboSNitches^31^. The receiver operating characteristic (ROC) curve was calculated based on the benchmark dataset to evaluate MeanDiff and EucDiff using different window sizes. The ROC curve plot was computed with the R package “pROC”^71^.

### Detection of riboSNitch-enriched or depleted elements

Cancer development is an evolutionary process, and as a vast number of somatic mutations are neutral mutations, we used mutation-profile-based random mutation procedure to simulate neutral mutations in cancer as the expected mutations. The neutral mutation rate can be calculated from intergenic or intron regions because such regions are less likely to be under the selective pressure than transcribed regions. Hence, in our study, we firstly computed the intronic mutation rate of each mutation type as the background mutation profile. As there were insufficient intronic mutations in the TCGA dataset, we calculated the intronic mutation frequency only in the ICGC dataset.

Considering different transcripts with different contexts, we also calculated the tri-nucleotide context of each transcript. To simulate neutral mutation in cancer, we used the cancer intronic mutation profile to represent the neutral mutation rate in cancer genome. Considering the mutation rate is related to mutation types and the sequence context, we taken the 5′ base and 3′base into account. Thus, we computed 96 possible mutation profile of intron (6 mutation types ∗ 4 types of 5′ base ∗ 4 types of 3′ base) and 32 corresponding tri-nucleotide composition of each transcript (2 types of base ∗ 4 types of 5′ base ∗ 4 types of 3′ base). Based on the intronic mutation frequency of 96 mutation types in ICGC dataset and trinucleotide distribution, we recalibrated mutation frequency of a specific transcript. After that, through simulating random sampling, we generated 1000 random mutations of each transcript. Then, we obtained the expected number of riboSNitches after using RNAplfold to calculate the RNA secondary structure and using MeanDiff and EucDiff to computed the difference of base pair probability between reference and mutated sequences. Similarly, the intersection of the top 2.5% mutations were considered as riboSNitches.

The number of riboSNitches in these 1000 randomizations was regarded as an expected value. In addition, we tally the riboSNitches and total mutations in the cancer mutation dataset as observed value. Mutations that occurred at the same position in different patients were counted independently. After the observed riboSNitches and expected number of riboSNitches were calculated, a one-sided Fisher’s exact test was conducted to identify genes that were significantly enriched or depleted the riboSNitches compared with the expected riboSNitches, and the resulting *P* value were corrected using the Benjamini and Hochberg method^42^ (Fig. 1). All statistical analyses were conducted in R. The results with a significant *P* value after the correction for the false discovery rate were regarded as putative riboSNitch-enriched or depleted candidates. Considering the complexity of coding region mutations, we only applied our approach to 5′UTRs, 3′UTRs as well as lncRNAs to discover riboSNitch-enriched or depleted candidates.

### Cancer-specific riboSNitch-enriched or depleted elements

To search for cancer-specific elements, we compared the riboSNitches in the cancer genome to in the 1000 Genome dataset. A one-sided Fisher’s exact test was used to determine whether riboSNitches were enriched in regions of the cancer genome. All elements with *P* value less than 10^−3^ were regarded as cancer-specific elements.

## Supplementary information

SUPPLEMENTARY INFO

Supplementary Table S1

Supplementary Table S2

Supplementary Table S3
