# Supplementary material for: Integrative Analysis of Somatic Mutations in Non-coding Regions Altering RNA Secondary Structures in Cancer Genomes
Source: Sci Rep. 2019 Jun 3;9:8205. doi: 10.1038/s41598-019-44489-5 (PMC6546760; doi:10.1038/s41598-019-44489-5)
Supplement: Supplementary file 1 — SUPPLEMENTARY INFO [file 41598_2019_44489_MOESM1_ESM.docx]

**Integrative Analysis of Somatic Mutations in Non-coding Regions Altering RNA Secondary Structures in Cancer Genomes**

Funan He^1^, Ran Wei^1^, Zhan Zhou^2^, Leihuan Huang^1^, Yinan Wang^1^, Jie Tang^1^, Yangyun Zou^1^, Leming Shi^1,3^, Xun Gu^4^, Melissa J. Davis^5^, Zhixi Su^1*‡^

^1^Ministry of Education Key Laboratory of Contemporary Anthropology, School of Life Sciences, Fudan University, Shanghai 200433, China

^2^Institute of Drug Metabolism and Pharmaceutical Analysis and Zhejiang Provincial Key Laboratory of Anti-Cancer Drug Research, College of Pharmaceutical Sciences, Zhejiang University, Hangzhou 310058, China

^3^Shanghai Cancer Center and Cancer Institute, Fudan University, Shanghai 200032, China

^4^Department of Genetics, Development and Cell Biology, Iowa State University, Ames, Iowa 50011, USA

^5^Bioinformatics Division, Walter and Eliza Hall Institute of Medical Research, 1G Royal Parade, Parkville, VIC 3052, Australia

***Correspondence to:** Zhixi Su, E-mail: suzhixi@gmail.com

^‡^ Present address: Singlera Genomics Inc, Shanghai, China

**Supplementary Information**

**Supplementary Fig. S1.** The MeanDiff distribution of different regions (5’UTR, 3’UTR, CDS, and lncRNAs) in the ICGC dataset.

**Supplementary Fig. S2.** The EucDiff distribution of different regions (5’UTR, 3’UTR, CDS, and lncRNAs) in the ICGC dataset.

**Supplementary Fig. S3.** The MeanDiff distribution of different regions (5’UTR, 3’UTR, CDS, and lncRNAs) in the TCGA dataset.

**Supplementary Fig. S4.** The EucDiff distribution of different regions (5’UTR, 3’UTR, CDS, and lncRNAs) in the TCGA dataset.

**Supplementary Table S1**. The somatic mutation and riboSNitch distribution of different cancer types in TCGA, ICGC and other previous publications dataset.

**Supplementary** **Table S2**. All the riboSNitch-enriched 5'UTR, 3’UTR, and lncRNA elements in ICGC dataset at the different cutoff.

**Supplementary Table S3**. All the riboSNitch- depleted 5'UTR, 3’UTR, and lncRNA elements in ICGC dataset at the different cutoff.

**Supplementary Fig. S1.**


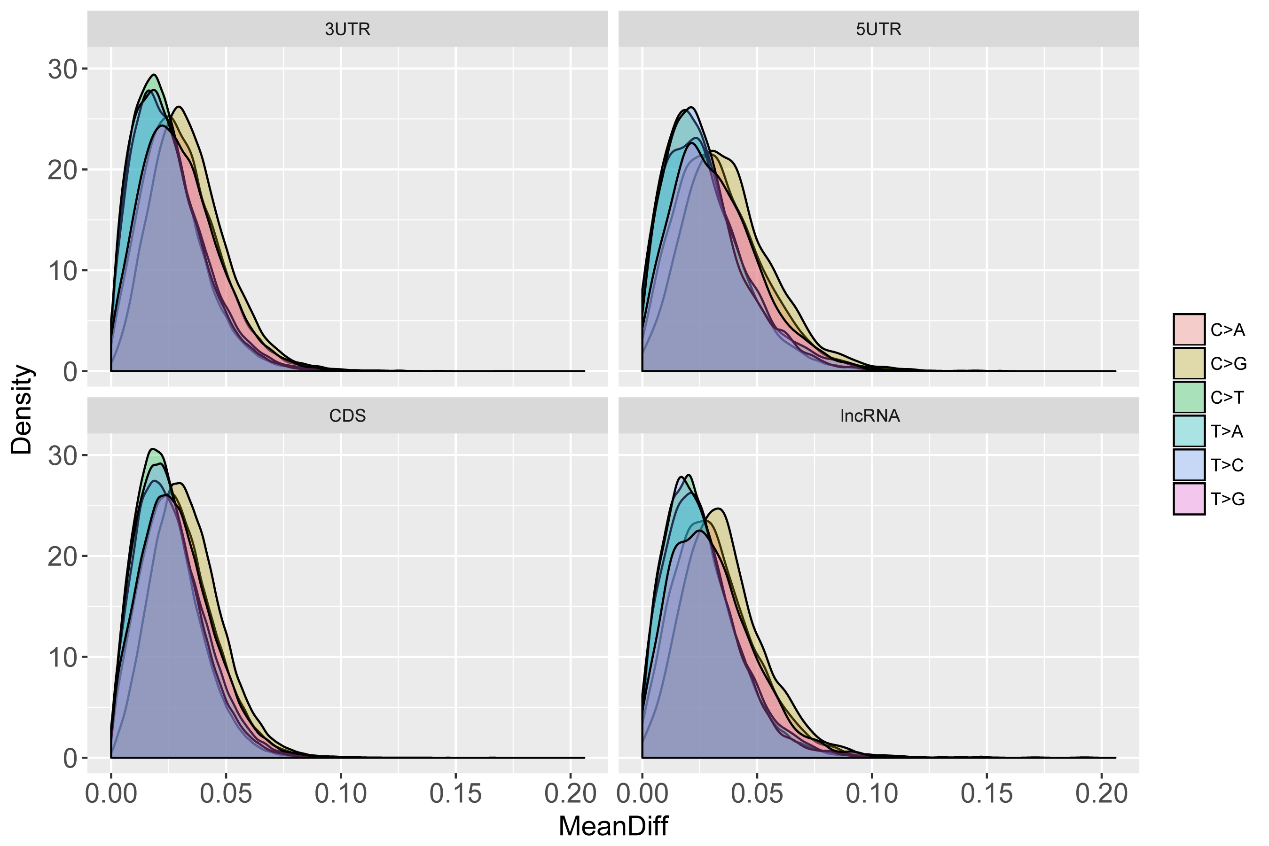


**Supplementary Fig. S2.**


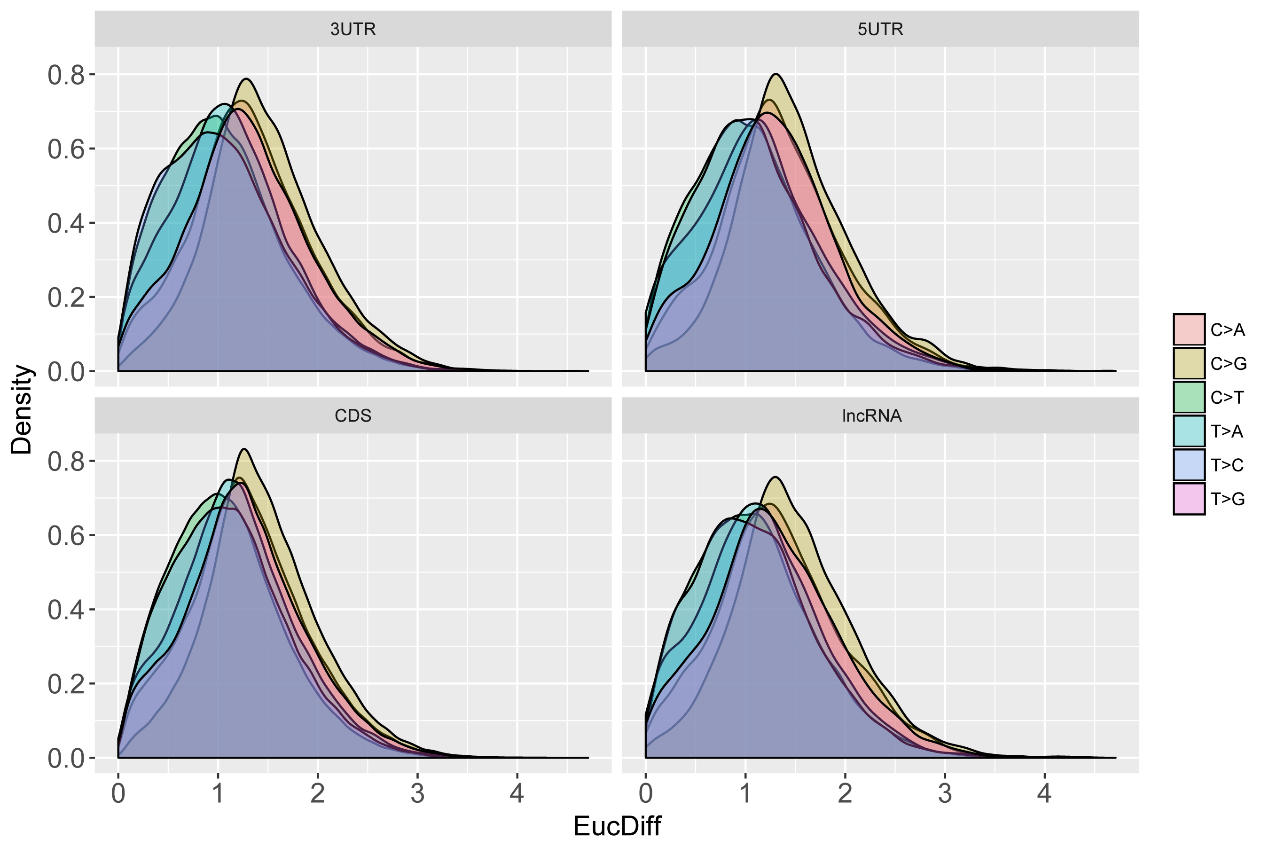


**Supplementary Fig. S3**

**
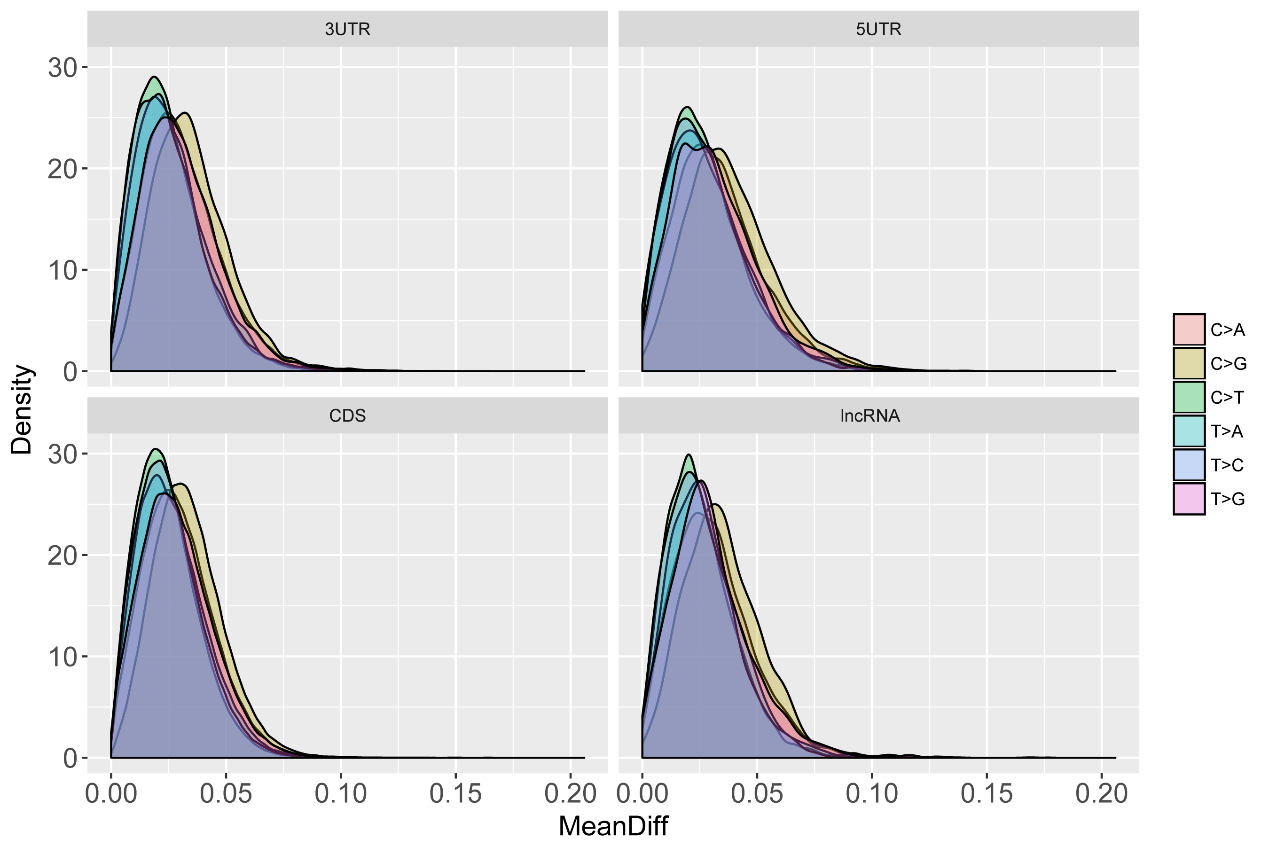
**

**Supplementary Fig. S4**

**
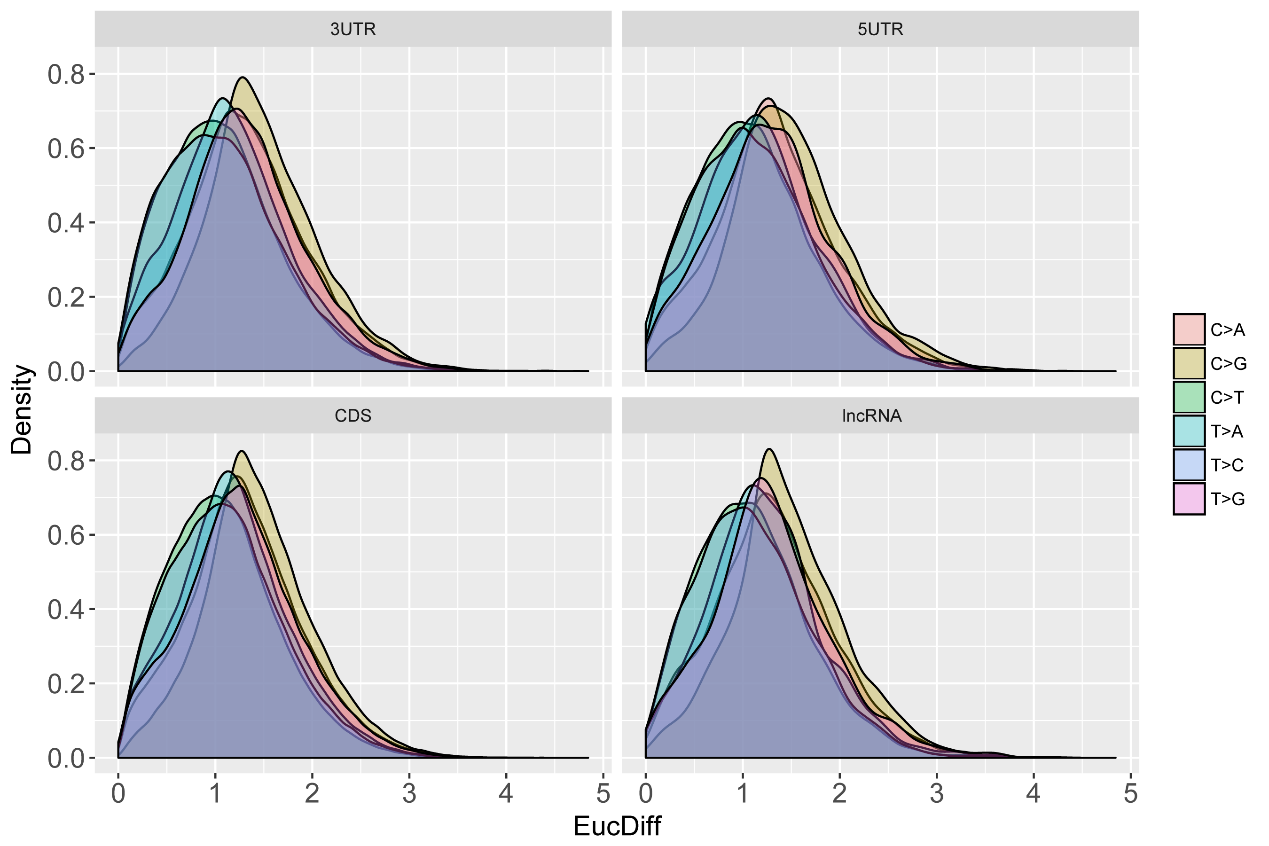
**
